# Interleukin-1 loop model for pathogenesis of Langerhans cell histiocytosis

**DOI:** 10.1186/s12964-015-0092-z

**Published:** 2015-02-22

**Authors:** Ichiro Murakami, Michiko Matsushita, Takeshi Iwasaki, Satoshi Kuwamoto, Masako Kato, Keiko Nagata, Yasushi Horie, Kazuhiko Hayashi, Toshihiko Imamura, Akira Morimoto, Shinsaku Imashuku, Jean Gogusev, Francis Jaubert, Katsuyoshi Takata, Takashi Oka, Tadashi Yoshino

**Affiliations:** Division of Molecular Pathology, Faculty of Medicine, Tottori University, 86 Nishi-cho, Yonago, 683-8503 Japan; Department of Pathobiological Science and Technology, School of Health Science, Faculty of Medicine, Tottori University, Yonago, 683-8503 Japan; Department of Pathology, Tottori University Hospital, Yonago, 683-8503 Japan; Department of Pediatrics, Kyoto Prefectural University of Medicine, Kyoto, 602-8566 Japan; Department of Pediatrics, Jichi Medical University School of Medicine, Shimotsuke, 329-0498 Japan; Division of Pediatrics and Hematology, Takasago-seibu Hospital, Takasago, 676-0812 Japan; Inserm U507 and U1016, Institut Cochin, Paris, 75014 France; University of Paris Descartes (Paris V), Paris, 75006 France; Department of Pathology, Okayama University Graduate School of Medicine, Dentistry and Pharmaceutical Sciences, Okayama, 700-8530 Japan

**Keywords:** Langerhans cell histiocytosis, Merkel cell polyomavirus, Interleukin 1, Toll-like receptor, Interleukin 17 receptor, Src homology 2 domain tyrosine phosphatase 1

## Abstract

We propose Langerhans cell histiocytosis (LCH) is an inflammatory process that is prolonged by mutations. We hypothesize that Merkel cell polyomavirus (MCPyV) infection triggers an interleukin-1 (IL-1) activation loop that underlies the pathogenesis of LCH. Langerhans cells (LCs) are antigen presenting cells in the skin. When LCs encounter exogenous antigens, they migrate from the epidermis into draining lymphoid tissues to initiate T-cell activity. It has been proposed that LC migration-related factors, including E-cadherin, matrix metalloproteinase, and Notch ligand induce LCH activity. We found that the tyrosine phosphatase SHP-1, which binds IL-1 receptor-associated kinase 1, is expressed at a significantly higher level in LCH affecting multiple organ systems (MS-LCH) than in LCH affecting a single organ system (SS-LCH). IL-1 stimulates T helper 17 cells and their signature cytokine IL-17 had been a matter of controversy. We detected higher levels of IL-17A receptor expression in MS-LCH than in SS-LCH and proposed an IL-17 endocrine model that could settle the controversy. IL-1 is the first cytokine secreted in response to sensitizers and promotes LC migration from sentinel tissues. Myeloid differentiation primary response 88 (MyD88), downstream of the IL-1 receptor, has functions in both RAS signaling and inflammation, leading to human cell transformation. In 2010, an activating mutation in the B-rapidly accelerated fibrosarcoma gene (*BRAF*) V600E was found in LCH. This *BRAF* mutation induces phosphorylation of the extracellular signal-regulated kinase (ERK) that may play an important role with MyD88 in LCH pathogenesis. However, phosphorylated ERK (pERK) is rapidly dephosphorylated by dual specificity phosphatase 6 (DUSP6), and limited proliferation is predicted in *BRAF* mutant cells. MyD88 binds pERK via its D-domain, thereby preventing pERK–DUSP6 interaction and maintaining ERK in an active, phosphorylated state. We detected MCPyV-DNA in the peripheral blood cells of two out of three patients with LCH in high-risk organs but not in those of patients with LCH in non–high-risk organs (0/12; *P* = .029). MCPyV infection can trigger precursor LCH cells with *BRAF* mutation to produce IL-1; the IL-1 loop is amplified in all LCH subclasses. Our model indicates both *BRAF* mutation and IL-1 loop regulation as potential therapeutic targets.

## Introduction

Langerhans cell (LC) histiocytosis (LCH) is characterized by the proliferation of CD1a-positive abnormal LC-like cells (LCH cells). LCH is classified by its involvement of either a single organ system (SS-LCH) or multiple organ systems (MS-LCH) [1]. The latter form is frequent in children younger than 2 years, whereas SS-LCH is more common in children older than 2 years [2,3]. This rare disease affects 4–9 children per million each year [4-6]. The liver, spleen, and bone marrow (BM) are considered high-risk organs for LCH [7,8]. Therefore, LCH is also classified as involving at least one high-risk organ [LCH-risk organ (RO) (+)] or a no high-risk organ [LCH-RO (−)] [7]. Though most patients with LCH-RO (+) develop MS-LCH, some patients with the involvement of only one high-risk organ have a milder case, with symptoms similar to those observed in SS-LCH [9,10]. The morphology of lesions is so uniform that pathologists cannot determine whether a given biopsy originates from a patient with SS-LCH or MS-LCH, from a patient with LCH-RO (+) or LCH-RO (−), or from a child or an adult [11]. However, the clinical course of LCH is remarkably variable, ranging from lesions that spontaneously resolve, to a chronic disease that can be widespread and sometimes lethal [12-15].

Although LCH was first described a century ago, the etiology is still not understood [16]. For decades, it was thought that the disease is a reactive disorder rather than a neoplastic process [16]. As the former name, “eosinophilic granuloma” indicates that lesional LCH morphology is reminiscent of tissue reactions to an intracellular pathogen, of which tuberculous granuloma is the prototype [11]. Scabies infections are reported to induce LC hyperplasia, which mimics LCH [17]. However, recent studies indicate that LCH has a more neoplastic character [18-20]. While unexpected remission can rarely occur in neoplasms, spontaneous healing is more common in LCH, suggesting that there may be multiple pathobiologic contributions to the LCH process [11,21,22]. For example, an epidemiologic study revealed that risk factors for MS-LCH include an increase in infections, the use of antibiotics in the first 6 months of life, and a family history of thyroid disease, whereas SS-LCH is significantly associated with diarrhea and vomiting in the postnatal period [23].

In this review, we propose a new model for LCH pathogenesis in which the disease is a reactive disorder with underlying neoplastic potential. In other words, LCH is an inflammatory process that is prolonged by mutations.

## Review

### Langerhans cells

**Figure 1:**
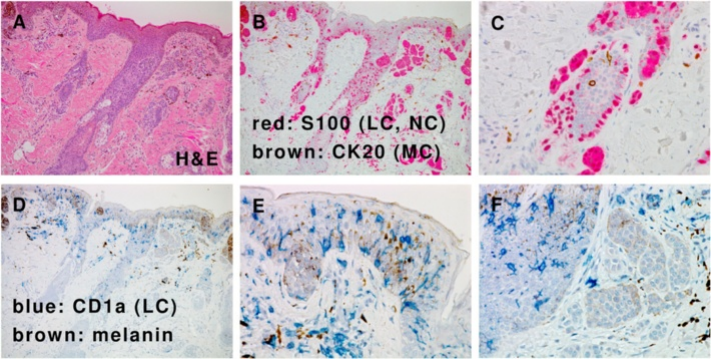
**Langerhans cells and Merkel cells in the epidermis. (A)** Skin of one-year-old female baby with nevus cell nevus. (Hematoxylin and Eosin (H&E) stain). **(B)** Immunohistochemistry shows S100+ immature Langerhans cell (LC; red), nevus cell (NC; red), and cytokeratin 20 (CK20) + Merkel cells (MC; brown). Same area of panel **A**. **(C)** S100+ immature LC and NC (red) and CK20+ MC (brown) in a hair follicle. High power view of the left central part of panel **B**. **(D)** Immunohistochemistry shows CD1a + LC (blue) and CD1a- NC. Brown pigments correspond to melanin. Same area of panel **A**. **(E)** Immature LCs in the epidermis. High power view of panel **D**. **(F)** Immature LCs in a hair follicle are positive for CD1a, while the NCs are negative for CD1a. High power view of panel **D**.

**Figure 2:**
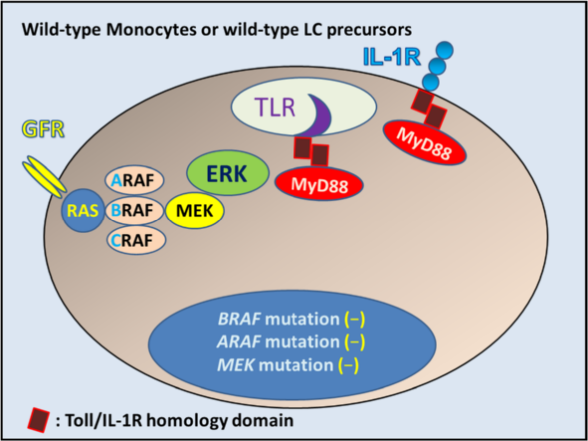
**Wild-type monocytes, wild-type LC precursors, or wild-type immature Langerhans cells without mutations.** In these cells, mitogens such as growth factors bind to and activate cell-surface receptors (GFR: growth factor receptor) that induce signaling through a complex consisting of adaptor proteins and exchange factors to activate RAS (Blue circle) on the inner surface of the cell membrane. Once activated, RAS binds to and activates the RAF family of proteins, including BRAF, which subsequently phosphorylates and activates MEK. Activated MEK subsequently phosphorylates and activates ERK. Activated ERK phosphorylates numerous substrates within the cytoplasm and nucleus, promoting cell division and enhancing survival, movement, and differentiation. In the case of Merkel cell polyomavirus (MCPyV) infection, some LCs may present MCPyV antigen, inducing adaptive immunity through Toll-like receptors (TLRs). IL-1 is the first cytokine secreted in response to sensitizers. IL-1 binds to IL-1 receptor (IL-1R) and promotes LC migration from sentinel tissue such as the skin. MCPyV interferes with LC function and maturation to evade immune surveillance, which might allow infection by inhibiting NF-κB essential modulator (NEMO) and down-regulation of TLR9.

In 1868, Langerhans [24] described a new epidermal cell type with dendritic extensions, which he believed to be part of the skin neural network [25]. Later named Langerhans cells (LCs), these cells differ from the Merkel cells that were first described in 1875 by Merkel as touch cells [26] (Figures 1 and 2). The function of LCs was unknown until 1973, when Steinman et al. [27,28] first discovered that dendritic cells (DCs) are in fact antigen presenting cells.

LCs are specialized immature DCs present in the skin (Figure 1). When LCs encounter exogenous antigens, they migrate from the epidermis to draining lymphoid tissues to initiate and present the major histocompatibility complex/peptide complexes to T cells. During this migration, LCs mature and the pattern of expression of their cell surface molecules changes [29,30]. LCs undergo the following steps for migration to the lymph nodes: down-regulation in the expression of E-cadherin, which anchors LCs to the epidermis; production of matrix metalloproteinase (MMP), which is required for passage through the basement membrane; an increase in the expression of the chemokine receptor CCR7, which guides migration toward CCL19 (MIP-3β) and CCL21 (SLC) [31]; and production of Notch ligands such as Delta and Jagged, which instruct distinct CD4 T helper cell fates [32].

**Figure 3:**
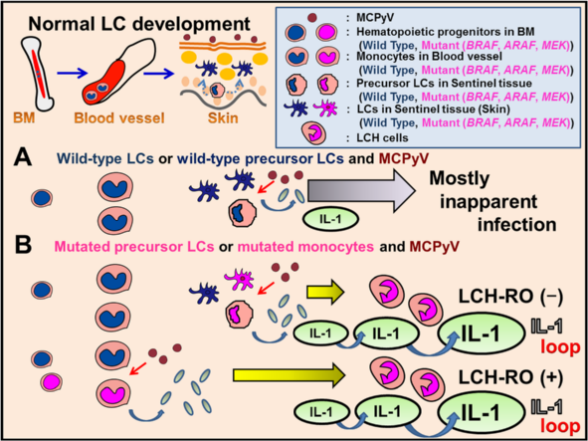
**Proposed relationship between MCPyV and wild-type Langerhans cell (LC) precursors and wild-type LCs or mutated monocytes and mutated LC precursors. (A)** MCPyV usually causes inapparent infection with immunoglobulin production against MCPyV, indicating acquired immunity against MCPyV is actuated by antigen presenting cells. LCs are antigen presenting cells derived from bone marrow (BM). LCs are normally generated and maintained locally in the steady state from precursors in the epidermis itself. In inflammation LC precursors are replenished by monocytes. Monocytes and LC precursors are candidate reservoir cells for MCPyV. **(B)** On the contrary, mutated precursor LCH cells (mutant monocytes, mutant LC precursors or mutant LCs) do not show inapparent infection against MCPyV; in such cases, a reactive disorder might be triggered, such as proliferation of LCH cells, cytokine storms including IL-1β, and clinical remissions. Mutated precursor LCH cells (mutant monocytes) in blood vessels recognize MCPyV and induce LCH-RO (+). Mutated LCH precursor cells (mutant LC precursors or mutant LCS) in peripheral tissues recognize MCPyV and induce LCH-RO (−). Most patients with LCH-RO (+) develop MS-LCH. Some patients with only one high-risk organ involved have a milder clinical course, similar to that observed in SS-LCH. In addition to IL-1 loop, serum level of IL-18 (one of IL-1 agonists) and osteopontin that is closely related to IL-1 levels were significantly higher in LCH-RO (+) than that found in LCH-RO (−).

LCs are normally generated and maintained locally in the steady state from precursors in the epidermis itself (Figure 3) [33]. This is sufficient to produce the low-level, steady-state efflux of LCs to the draining lymph nodes [33]. In mice, replenishment of LCs from bone-marrow precursors can only be observed experimentally following depletion of the LC population by local skin inflammation [33]. Then, the LC precursors are replenished by inflammatory monocytes that enter the epidermis from the bloodstream [33]. This ‘emergency’ replenishment of LCs might also be a model for the origin of LCs from non-inflammatory blood monocytes during early development, and perhaps a model for an ongoing, low-frequency event in the steady state [33].

LCH activity is controlled by enzymes and cytokines that are involved in LC migration and antigen presentation [34-40]. LCH is characterized by a lesional cytokine storm, the prominent cytokine sources being both LCH cells and T cells [16]. Interleukin (IL)-1, IL-18, and tumor necrosis factor-alpha (TNF-α) are important cytokines that promote LC migration from the skin [41-43]. LCs respond to many chemokines, in particular CCL20, which appears to be the most powerful chemokine to induce migration [16,44]. During pathogen invasion, immature LCs expressing CCR6, the major functional CCL20 receptor, would be attracted to the site of inflammation. After antigen uptake, maturation of LCs results in downregulation of CCR6 and expression of CCR7, resulting in attraction to CCL19 and CCL21 which are expressed in the T-zones of lymph nodes [45]. Flemming et al. [46] reported coincident expression of both CCR6 and CCR7 by LCH cells. MMPs such as MMP-1 [47] and MMP-9 [48,49] are also important to migration of LCs and are expressed in LCH cells (GSE16395) [50]. LCs and LCH cells can be recognized using electron microscopy by the presence of specific Birbeck granules [1], and by immunohistochemical staining with antibodies recognizing langerin (CD207) or CD1a [51]. Immature LCs are phagocytic cells, and the calcium-dependent (C-type) lectin langerin (CD207) plays a role in antigen capture and subsequent Birbeck-granule formation [52].

Although some LCH cells have close relationship with leukemia/lymphoma cells [53,54], origins of LCH cells were not determined. Generally it is not easy to conclude cell origins of neoplastic cells even by immunoglobulin productions [55]. Allen et al. [50] and Hutter et al. [40] tried to conclude cell lineages or origins from transcription profiles of LCH cells. Cultured monocytes dramatically change their characters by adding factors such as a conditioned medium of an established LCH lesion cell line [56]. Very recently high-throughput single cell transcriptomics revealed different temporal heterogeneity profiles among identical mouse bone-marrow-derived DCs after same stimulation [57]. So it is not easy to conclude LCH cell lineages under the cytokine storm [16] using transcription profiles. So we use the terms mutant monocytes and mutant precursor LCs as precursor LCH cells in this review as shown in Figure 3.

### Interleukin-1 autocrine/paracrine loop in LCH cells

**Figure 4:**
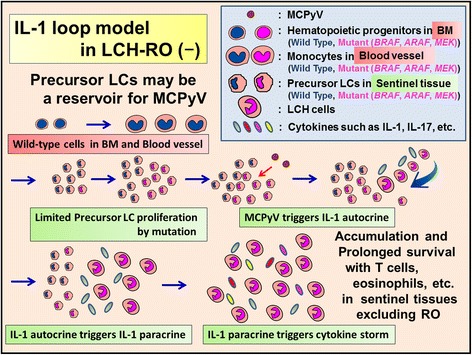
**Proposed relationship between MCPyV and mutated precursor LCs in LCH-risk organ (RO) (−).** Mutated LCH precursor cells (mutant LC precursors) in peripheral tissues recognize MCPyV and induce LCH-RO (−). LCH lesion is consisted by accumulation and prolonged survival of LCH cells with T cells, eosinophils, macrophages, etc. In LCH lesions the prominent sources of cytokine storm are T cells and LCH cells. LCH cells produce pro-inflammatory cytokines such as IL-1, anti-inflammatory cytokines such as IL-10, and growth factors. After accumulation and prolonged survival of LCH cells, LCH lesions diminish and spontaneous healing may be observed.

**Figure 5:**
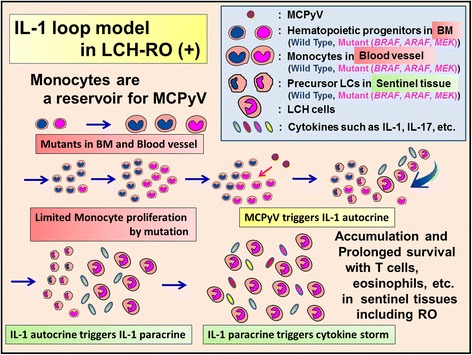
**Proposed relationship between MCPyV and mutated monocytes in LCH-RO (+).** Mutated precursor LCH cells (mutant monocytes) in blood vessels recognize MCPyV and induce LCH-RO (+). LCH lesion is consisted by accumulation and prolonged survival of LCH cells with T cells, eosinophils, macrophages, etc., which is not different from that of LCH-RO (−). In LCH lesions the prominent sources of cytokine storm are T cells and LCH cells. LCH cells produce pro-inflammatory cytokines such as IL-1, anti-inflammatory cytokines such as IL-10, and growth factors. After accumulation and prolonged survival of LCH cells, LCH lesions diminish and spontaneous healing may be observed. But circulating monocytes with mutation triggered by MCPyV caused disseminated LCH lesions including RO.

**Figure 6:**
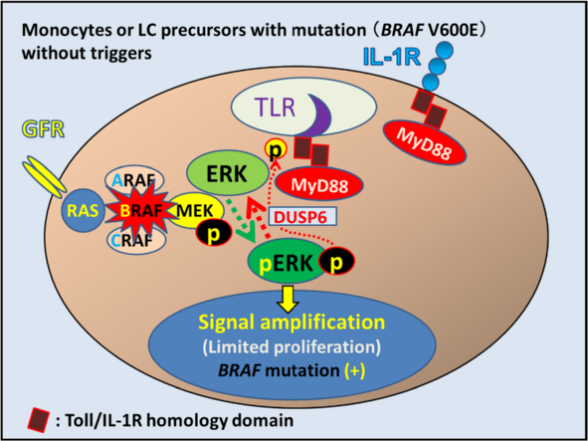
**Potential consequences of mutant** ***BRAF*** **V600E in LCH.** In mutant monocytes or mutant LC precursors, the constitutively active *BRAF* V600E mutant protein is predicted to bypass the requirement for mitogen-induced activation of RAF by RAS. The identification of activating *BRAF* mutations supports the hypothesis that LCH is a neoplastic process (oncogenic potential). However, phosphorylated ERK is rapidly dephosphorylated by DUSP6, which is constitutively expressed in LCH cells (GSE16395). Other factors, such as accumulated gene mutations and an inflammatory trigger of the RAS/RAF/MEK/ERK signaling pathway, thus appear to be involved in LCH pathogenesis. DUSP6: dual specificity phosphatase 6.

**Figure 7:**
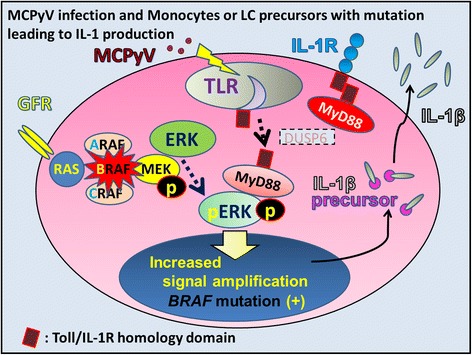
**Merkel cell polyomavirus is one candidate IL-1 trigger in LCH.** Merkel cell polyomavirus (MCPyV) may be detected by Toll-like receptors (TLRs). MyD88 is a TLR adaptor protein that binds to pERK, maintaining ERK in an active, phosphorylated state for a longer period. Activated ERK phosphorylates numerous substrates related to the expression of soluble mediators such as IL-1β. Because of the low viral load of MCPyV-DNA in LCH tissue, MCPyV does not seem to play an oncogenic role in LCH pathogenesis. MCPyV is regarded as a potential trigger of IL-1β production. Although MyD88 usually allows the activation of NF-κB, MCPyV might interfere with NF-κB activation by targeting NF-κB essential modulator (NEMO). IL-1β is synthesized as an inactive pro-form (IL-1β precursor) that accumulates in the cytosol. Cleavage of IL-1β precursor into active form requires the activation of inflammasomes.

**Figure 8:**
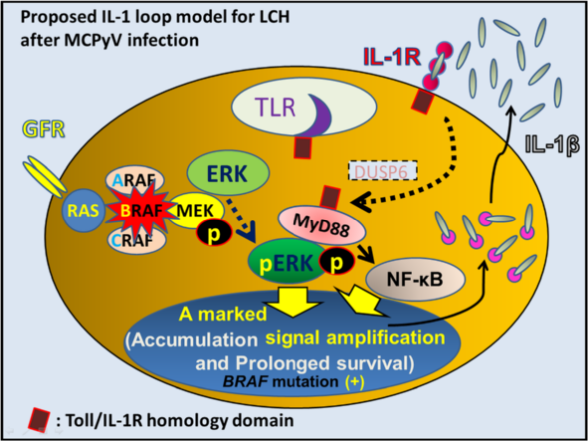
**Proposed IL-1-loop model in LCH as a reactive disorder triggered by MCPyV.** MyD88 is an adaptor of IL-1R and binds to pERK, maintaining ERK in an active, phosphorylated state. MyD88 also allows the activation of NF-κB, leading to the activation of further inflammatory and mitogenic signals. Induction of this IL-1β autocrine loop after MCPyV infection may lead to enhanced cell activation, proliferation, and eventually, transformation of LCH. In absence of MCPyV infection, the IL-1β paracrine loop also leads to enhanced cell activation, proliferation, and eventually, accumulation and cell survival of LCH cells. The clinical course of LCH may also be influenced by anti-inflammatory cytokines produced by T-cells under different conditions, including innate immunity alone and actuated acquired immunity against MCPyV.

The discovery of the B-Rapidly Accelerated Fibrosarcoma gene (*BRAF*) mutation in 2010 [20] gave new insights into LCH pathogenesis (Figures 3, 4, 5, 6, 7, and 8) and led to the hypothesis that the lesional IL-1 autocrine/paracrine loop [58,59] plays a major role in LCH pathogenesis as shown in Figures 3, 4, and 5 (IL-1 loop model).

**Figure 9:**
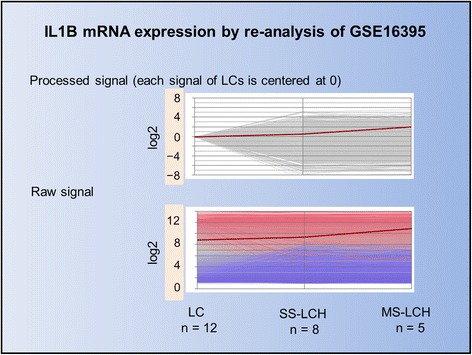
**Line Graph of IL1B mRNA data from GSE16395.** We compared GSE16395 mRNA array data between Langerhans cells (LCs) and LCH cells using the Subio platform. Each line represents a measured value. The red line represents IL1B mRNA expression, which is lower in both LCs and LCH cells according to the intensity of processed signals. However, using raw data signals, IL1B mRNA expression is high in both LCs and LCH cells. This phenomenon indicates that even slight stimuli, such as low temperature or tissue treatment using trypsin for LC isolation from the epidermis, can trigger IL-1β production. As previously reported, IL-1β expression was indicated. In addition IL-1β expression was higher in MS-LCH than SS-LCH.

The antigen-presentation capabilities of LCs are revealed only after IL-1β– and TNF-α–induced migration toward skin-draining lymph nodes [60,61]. LCH cells produce high levels of multiple cytokines, including IL-1 [62,63]. IL-1β is the first cytokine secreted in response to topical allergens via the inflammasome [64]; IL-1β mRNA can be detected in LCs as early as 15 min after exposure to sensitizers [65]. Although IL-1β is not produced under normal conditions, it is easily induced by slight stimulation, as shown by studies at the mRNA level [50]. Purification of LCs (Figure 1) from 4% of the entire cell population in the epidermis [66] to 97.3% [50] using anti-CD207 antibody was performed after incubation in RPMI 1640 with dispase II at 4°C for 8 h and 0.25% trypsin-EDTA for 15 min. Transient Receptor Potential (TRP) channels [67,68] are sensitive to temperature [69,70] and induce inflammasome activation [71]. In addition, the CD1a molecule is sensitive to trypsin [72]. Thus, this purification can induce LCs to produce IL-1β by comparing mean raw signals of IL-1β mRNA (log2) as follows: 8.8698 (LCs, n = 12), 9.379 (LCH cells of SS-LCH, n = 8), and 10.8729 (LCH cells of MS-LCH, n = 5) by re-analyses of GSE16395 [50] using Subio platform (http://www.subio.jp/products/platform) [39] (Figure 9). IL-1 stimulates MyD88, which activates nuclear factor-κB (NF-κB), leading to the subsequent activation of further inflammatory and mitogenic signals [73].

Based on the serum and saliva levels of IL-1 [74,75], we advocate that the lesional IL-1 autocrine/paracrine loop [58,59] plays an important role in LCH pathogenesis, as shown in Figures 3, 4, and 5 (IL-1 loop model). This combination of MyD88-dependent signals may lead to enhanced cell activation, proliferation, and eventually, accumulation and prolonged cell survival [58,73] of LCH lesions.

### Cigarette smoke and IL-1

Pulmonary LCH predominantly affects young adults and occurs almost exclusively in smokers [76-79]. Studies of cigarette smoke-induced accumulation of lung DCs in mice indicate an IL-1–dependent phenomenon [80]. In addition, cigarette smoke-induced pulmonary inflammation is dependent on Toll-like receptor (TLR) 4/MyD88 and IL-1R1/MyD88 signaling [81].

### Merkel cell polyomavirus is a candidate for triggering the IL-1 loop involved in LCH pathogenesis

Patients with LCH often have dermal disorders such as seborrheic dermatitis [7] concomitant to LCH lesions [82], preceding LCH lesions [83-85], or following LCH lesions [86]. Perianal lesions [87,88] or a lesion on the soles [89] were also reported. Stein et al. [90] reported that children who present with LCH from birth to 4 weeks of age are not diagnosed with LCH until an average of 3.5 months of age because the eruptions are nonspecific in nature [91]. We recently described the possibility of a causal relationship between LCH and dermotropic Merkel cell polyomavirus (MCPyV) [92], which was discovered as the major pathogenic agent in Merkel cell carcinoma of the skin in 2008 [93]. Our data indicate that MCPyV-DNA sequences are present in LCH tissues (12/13) excluding pulmonary LCH, with significant differences between LCH tissues and controls that included patients with dermatopathic lymphadenopathy (5/20; *P* = .0002) and reactive lymphoid hyperplasia (0/5; *P* = .0007) [92]. The numbers of MCPyV DNA sequences in all four LCH tissues from patients younger than 2 years indicated a significant difference from tissues of non-LCH dermal disease patients of the same age (0/11; *P* = .0007) [92]. Our data suggest that LCH is a reactive disorder with an underlying oncogenic potential. Thus, both LCH and pulmonary LCH harbor the *BRAF* V600E mutation [20,94] and appear related to stimuli such as viral infection [92,95,96] and cigarette smoking [97,98]. In addition, the removal of stimuli is reported to cause spontaneous healing of LCH [99,100].

Expression of the constitutively active *BRAF* V600E mutant in LCH cells is predicted to bypass the requirement for mitogen-induced activation of RAF by RAS (Figure 6) [20,101]. The identification of activating *BRAF* mutations supports the hypothesis that LCH is a process with oncogenic potential [20]. A mouse LCH model using a *BRAF* V600E construct under control of the CD11c promoter and a *BRAF* V600E construct under control of the langerin promoter indicates that the *BRAF* V600E is not only a marker but also an essential driver of LCH pathogenesis [102]. Moreover, phosphorylated extracellular signal-regulated kinase (ERK) (pERK) is rapidly dephosphorylated by dual specificity phosphatase 6 (DUSP6) [73,103], which is overexpressed in LCH cells [50]. However, *BRAF* V600E mutations are also detected in non-neoplastic disorders such as nevus cell nevus (Figure 1) [104] and hyperplastic polyps of the colon [105]. Thus, LCH pathogenesis requires both limited proliferation of precursor LCH cells harboring the *BRAF* V600E mutation and the accumulation of gene mutations or an inflammatory trigger that activates the RAS/RAF/mitogen-activated protein kinase kinase (MEK)/ERK signaling pathway [101].

We reported the presence of MCPyV-DNA in the peripheral blood cells of two out of three patients with LCH-RO (+) but not in the blood cells of 12 of 12 patients with LCH-RO (−) (*P* = .029) [92]. Berres et al. [102] reported that patients with LCH-RO (+) carried the *BRAF* V600E mutation in circulating CD11c + and CD14+ cellular fractions as well as in BM CD34+ hematopoietic cell progenitors, whereas the mutation was restricted to lesional LCH cells in patients with LCH-RO (−). These findings specifically observed in LCH-RO (+) suggest the LCH pathogenetic pathway shown in Figures 3, 4, and 5, though it needs further confirmation to conclude.

MCPyV interferes with DC function (Figures 2 and 3) to evade immune surveillance by targeting a NF-κB essential modulator (NEMO) [106] and down-regulating TLR9 [107]. Exposure to MCPyV as measured by serum antibodies against the viral capsid proteins appears to be widely prevalent among healthy subjects [108,109]. Inapparent existence of MCPyV is indicated on the skin and environmental surface [110,111]. Pancaldi et al. [112] indicated buffy coats of healthy adult blood donors, which were examined for MCPyV DNA tag sequences, showed a prevalence of 22%, with viral loads ranging from 10 to 100 molecules per 100 000 cells (0.0001 to 0.001 per cell). Mertz et al. [113] reported that CD14 + CD16− inflammatory monocytes are a reservoir for MCPyV, but CD14^lo^CD16+ resident monocytes, lymphocytes, or granulocytes are not. Our data from micro-dissected LC in both dermatopathic lymphadenopathy [92] and LC sarcoma [114] suggest that monocytes, precursor LCs, or LCs are one of the reservoir cells for MCPyV (Figure 3). Members of the TLR/IL-1 receptor (IL-1R) superfamily play a fundamental role in the immune response [115]. Viral “pathogen-associated molecular patterns” are recognized by specific TLRs [116]. TLR agonists stimulate IL-1β production in DC [117], where TLR-triggered ERK activation play important roles [118]. IL-1α expression is induced by TLR-mediated NF-κB activation; such activation has been observed in some LCH cases [119,120], with/without the presence of IL-1β [121]. All TLRs except TLR3 use the common MyD88-dependent pathway [122]. MyD88 is one of the adaptor proteins that links TLR/IL-1R [123] and binds to pERK via its D-domain, thereby preventing pERK–DUSP6 interaction and maintaining ERK in an active, phosphorylated state for a longer period [73]. This MyD88-dependent signal may lead to enhanced cell activation, proliferation, and eventually, accumulation and prolonged survival [58,73] of a given LCH lesion (Figure 8).

### IL-1 family

The IL-1 family includes seven ligands with agonist activity (IL-1α, IL-1β, IL-18, IL-33, IL-36α, IL-36β, and IL-36γ), three receptor antagonists (IL-1Ra, IL-36Ra, and IL-38), and an anti-inflammatory cytokine (IL-37). Members of the IL-1R family include six receptor chains that form four signaling receptor complexes, two decoy receptors (IL-1R2 and IL-18BP), and two negative regulators (TIR8 or SIGIRR, and IL-1RAcPb). A tight regulation via receptor antagonists, decoy receptors, and signaling inhibitors ensures a balance between amplification of innate immunity and uncontrolled inflammation [124].

Our group reported that the serum level of IL-1α was significantly higher in patients with LCH than in controls [125], which suggests that IL-1 endocrine loop also plays an important role in LCH, in addition to the lesional IL-1 autocrine/paracrine loop. Serum levels of IL-18 were reported as significantly higher in LCH-RO (+) than in LCH-RO (−) [125]. Coury et al. [74] reported that serum levels of IL-1β in patients with LCH were not high; however, Preliasco et al. [75] reported IL-1β was increased in the saliva of children with LCH. Rosso et al. [126] reported that serum levels of IL-1Ra were significantly higher in patients with LCH than in controls. A tight regulation of cytokines via receptor antagonists such as IL-1Ra ensures a balance between amplification of innate immunity and uncontrolled inflammation. This balance may be overwhelmed by the cytokine storm caused by amplification of the IL-1 loop in *BRAF* mutant cells detected in patients with LCH (Figures 4 and 5). Agents inducing both IL-1β and IL-1Ra are viruses, bacteria, yeasts, soluble microbial products, IL-1, and TNF [127].

IL-1-mediated inflammation is also proposed to contribute to the development and progression of some cancers [73], such as melanoma [128,129]. The oncogenic *BRAF* V600E mutation promotes stromal cell-mediated immunosuppression via induction of IL-1 in melanoma [130]. Incisional biopsy of melanoma of the head and neck, which has a significant statistical association in the development of metastasis as compared to excisional biopsy [131], might be related to IL-1 expression through TRP channel triggered by mechanical stimulation [132,133]. IL-18 expression is also related to cancer-induced immunosuppression [134]. The following gene expressions are induced by IL-1: cytokines (TNF, IL-2, IL-3, IL-6, IL-12, GM-CSF, TGFβ, etc.); cytokine receptors (for IL-2, for IL-3, for IL-5, for GM-CSF, for c-kit); proinflammatory mediators (cyclooxygenase, etc.); hepatic acute phase reactants, clotting factors (fibrinogen, plasminogen activator inhibitor, etc.); oncogenes (c-*jun*, c-*abl*, c-*fms*, c-*myc*, c-*fos*) [127].

### The Src homology region 2 domain-containing phosphatase −1 (SHP-1)

The tyrosine phosphatase SHP-1 plays an important role in DCs [135]. We thus investigated SHP-1 levels in LCH and found significantly higher expression of SHP-1 in MS-LCH than that in SS-LCH [38]. From this report, it has been proposed that SHP-1 might promote IL-1R/TLR–activated production of type I interferon, which is one of innate antiviral cytokines [136], by inhibiting IL-1R associated kinase 1 [137].

### IL-17A receptor (IL-17RA)

Ziegler-Heitbrock et al. [138] proposed three types of monocytes: classical CD14++CD16− monocytes; intermediate CD14++CD16+ monocytes; nonclassical CD14 + CD16++ monocytes. Mertz et al. [113] reported that CD14 + CD16− inflammatory monocytes are a reservoir for MCPyV; they noted that circulating monocytes caused disseminated psoriatic lesion, in which IL-1 may be a key inflammatory mediator [139-141], over a course of 7 years in one patient who subsequently developed Merkel cell carcinoma. They [113] reported MCPyV DNA in a psoriatic lesion, too. Psoriasis is a skin disorder in which T lymphocytes and DCs play a central role [142]. IL-1 is a key mediator of psoriasis [139]. Viral infection such as vesicular stomatitis virus, vaccinia virus, and a variety of influenza A viruses triggers rapid differentiation of human blood monocytes into DCs [143]. Monocytes as a reservoir for MCPyV might have a potential to differentiate into DCs. T helper 17 cells (Th17) and their signature cytokine IL-17 have a critical role in the pathogenesis of psoriatic disease [144,145]. TLR2-activated human LCs promote Th17 polarization via IL-1β, TGF-β, and IL-23 [146] through MyD88 [147]. These data may suggest MCPyV could promote IL-1β via TLR of LCs. Inhibition of ERK was observed to suppress IL-1 and IL-23 production by DCs [118]. IL-1α and IL-6, which also stimulate Th17, were reported as significantly higher in LCH tissues [3,62,148,149] and cultured cells from LCH lesion [150], than in controls (*P* < 0.05) [125]. We detected higher levels of IL-17RA expression in MS-LCH than in SS-LCH [39] and proposed an IL-17 endocrine model that could settle the IL-17A controversy and the IL-17A paradox [74,151,152] in LCH pathogenesis.

### Innate and adaptive immunity against MCPyV

As shown in Figures 3, 4, and 5, we propose different MCPyV infection patterns between LCH-RO (+) and LCH-RO (−) [92]. These patterns differ with respect to the presence or absence, respectively, of cells carrying the mutated *BRAF* V600E in BM or among circulating fractions [102]. We have observed that IL-18 serum levels are higher in LCH-RO (+) than in LCH-RO (−) [125]. IL-18 induces LC migration via TNF-α and IL-1β molecules [153]. Seroepidemiological surveys in Cameroon report MCPyV seroprevalence rates in children 1 year old and younger as 60% (0–2 months, n = 34), 70% (3–4 months, n = 11), 35% (5–10 months, n = 49), and 20% (11–12 months, n = 23) [154]. In contrast, the MCPyV seroprevalence rates in LCH patients aged 1 month to 1 year were 0% (n = 6), although the seroprevalence rates among LCH patients ≥2 years of age are similar to that of other groups [108,109]. Primary infection by MCPyV without maternal immunoglobulin against MCPyV might play a significant pathogenic role in LCH among patients aged 1 month to 1 year, when MS-LCH or LCH-RO (+) often occurs [2,3]. Innate immune function between newborns and elderly is extremely different and large quantities of IL-6 and IL-23 after TLR stimulation by term newborns are indicated [155]. Thus, IL-6 amplifier activation may also influence the clinical course of LCH [156]. Kumar et al. [157] found that MCPyV-specific Th-cells secrete the Th2-like cytokine IL-13, the regulatory cytokine IL-10 (anti-inflammatory cytokine), and the Th1-like cytokine IFN-γ (a major antiviral cytokine). IL-13 induces IL-1 Ra [158] whose levels in sera of LCH patients are reported to be high [126]. IL-10 prevents LC maturation and thought to contribute to the maintenance of LCH cells in an immature stage of differentiation [34]. The levels of these molecules were significantly higher among MCPyV-seropositive subjects than among seronegative subjects [157]. Senechal et al. [12] pointed out that compared with controls, the expansion of regulatory T-cells, which are inhibitory in adaptive immunity, is observed in both LCH lesion and peripheral blood of patients with LCH. Immune conditions including the release of regulatory T-cells and anti-inflammatory cytokines might modify the balance between amplification of innate immunity against MCPyV and subsequent uncontrolled inflammation.

Both innate and acquired immunity are regulated by the coordination of many molecules, including osteopontin (OPN) [159,160]. Expression of the OPN protein, encoded by *SPP1* [50], is closely related to IL-1 levels [161-164]. We have recently reported high serum levels of OPN in LCH-RO (+) [165].

### RAS/RAF/MEK/ERK signaling pathway in LCH cells

*BRAF* V600E mutations are present in 57% of all patients with LCH [20]. pERK was detected in LCH without the *BRAF* V600E mutation [20], indicating that other factors in the RAS/RAF/MEK/ERK signaling pathway may also be important in LCH pathogenesis. H-ras overexpression was first reported in LCH [166]. In cases of LCH without the *BRAF* V600E mutation, other mutations have been reported, including ARAF (one patient of two *BRAF* wild-type cases) [167] and MAP2K1 (MEK1) mutations [7/21 (33%) to 11/22 (50%) of *BRAF* wild-type cases] [168,169]. Very recently, Chacraborty et al. [169] reported an ERBB3 mutation.

Berres et al. [102] reported that hematopoietic progenitor cells are linked not only to LCH but also to juvenile xanthogranuloma or Erdheim–Chester disease (ECD), suggesting a common denominator for these conditions. Hervier et al. [170] also pointed out a relationship between LCH and ECD. NRAS mutations were detected in 3/17 ECD *BRAF* V600E wild-type patients, and PIK3CA mutations were detected in 7/55 patients, of whom 4 also had *BRAF* mutations [171]. As for cytokines, several studies supported a central role of the IL-1 network in ECD [172,173].

### Other triggers of the IL-1 loop in LCH

Many factors including infectious agents and stress factors, inflammatory substances, and clotting factors are inducers of IL-1 [127]. In addition to MCPyV and cigarette smoking, Bacillus Calmette-Guerin (BCG) [174] and Epstein–Barr virus (EBV) infection [95,96] have been indicated as deeply involved in LCH pathogenesis. Induction of IL-1 by BCG [175-177] or EBV infection [178] was indicated.

## Conclusions

We propose a new model for LCH pathogenesis in which the disease is a reactive disorder with underlying neoplastic potential. In other words, LCH is an inflammatory process that is prolonged by *BRAF* mutation. Spontaneous healing is more common in LCH and diminished activity of LCH cells might not be reversed by any more IL-1 at the later stage as shown in experiments using cultured LCH cells [179]. Triggers such as MCPyV stimulate cells with *BRAF* V600E mutations to produce IL-1 and a subsequent IL-1 autocrine/paracrine/endocrine loop cytokine storm. The state of acquired immunity against MCPyV may also influence the clinical course of LCH.

Together, these data indicate the importance of *BRAF* V600E mutations [180,181] as well as suggest that IL-1 could serve as a therapeutic target for LCH treatment, especially for prolonged inflammatory process supplied from circulating mutated monocytes in LCH-RO (+) [182]. IL-1R antagonists such as anakinra, already tried for ECD treatment [183,184], rilonacept, and canakinumab, with taking care of serious unwanted side-effects, might interrupt the out-of-control IL-1 loop, providing a therapeutic strategy for LCH treatment.

## Abbreviations

BM: Bone marrow
*BRAF*: B-rapidlly accelerated fibrosarcoma gene
DC: Dendritic cell
DUSP6: Dual specificity phosphatase 6
ECD: Erdheim-Chester disease
ERK: Extracellular signal-regulated kinase
GFR: Growth factor receptor
IL-1: Interleukin 1
IL-1R: IL-1 receptor
IL-1Ra: IL-1 receptor antagonist
IL-17RA: IL-17A receptor
LCs: Langerhans cells
LCH: Langerhans cell histiocytosis
LCH cells: CD1a-positive abnormal LC-like cells
LCH- RO (+): LCH developed in at least one high-risk organ
LCH-RO (−): LCH without high-risk organ involvement
MCPyV: Merkel cell polyomavirus
MEK: mitogen–activated protein kinase kinase
MMP: Matrix metalloproteinase
MS-LCH: Multisystem LCH
MyD: Myeloid differentiation primary response
NEMO: Nuclear factor-κB essential modulator
NF-κB: Nuclear factor-κB
OPN: Osteopontin
pERK: phosphorylated ERK
SS-LCH: Single-system LCH
Th: T helper
TNF-α: Tumor necrosis factor-alpha
TLR: Toll-like receptor
TRP: Transient Receptor Potential
